# Bis(di-2-pyridylmethane­diol-κ^3^ *N*,*O*,*N*′)nickel(II) dinitrate

**DOI:** 10.1107/S1600536809018728

**Published:** 2009-05-23

**Authors:** Seung Man Yu, Young Joo Song, Kang Chul Kim, Cheal Kim, Youngmee Kim

**Affiliations:** a Department of Fine Chemistry and Eco-Product and Materials Education Center, Seoul National University of Technology, Seoul 139-743, Republic of Korea; b Department of Computer Engineering, Yeosu Campus, Chonnam National University, Yeosu 550-749, Republic of Korea; c Department of Chemistry and Nano Science, Ewha Women’s University, Seoul 120-750, Republic of Korea

## Abstract

The title compound, [Ni(C_11_H_10_N_2_O_2_)_2_](NO_3_)_2_, consists of an Ni^II^ atom coordinated by two tridentate chelating di-2-pyridylmethane­diol [(2-py)_2_C(OH)_2_] ligands. The Ni^II^ atom is located on an inversion center. The geometry around the Ni^II^ atom is distorted octa­hedral. The *gem*-diol (2-py)_2_C(OH)_2_ ligand adopts the coordination mode η^1^:η^1^:η^1^. The Ni—N and Ni—O bond lengths are typical for high-spin Ni^II^ in an octa­hedral environment [Ni—N = 2.094 (2) and 2.124 (3) Å, and Ni—O = 2.108 (3) Å]. One of the hydr­oxy H atoms is split over two positions which both inter­act with the nitrate anion. The occurence of different O—H⋯O hydrogen bonds leads to the formation of a layer parallel to the (101) plane.

## Related literature

For background information, see: Efthymiou *et al.* (2006 ▶); Moragues-Cánovas *et al.* (2004 ▶); Papaefstathiou & Perlepes (2002 ▶); Papatriantafyllopoulou *et al.* (2007 ▶); Stoumpos *et al.* (2008 ▶, 2009 ▶). For related structures, see: Li *et al.* (2005 ▶); Wang *et al.* (1986 ▶).
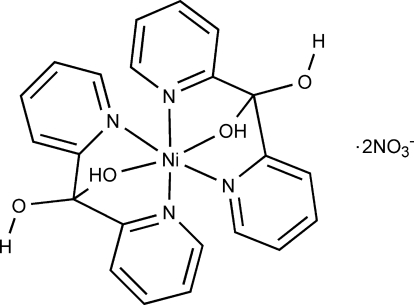


## Experimental

### 

#### Crystal data

[Ni(C_11_H_10_N_2_O_2_)_2_](NO_3_)_2_
*M* *_r_* = 587.15
Monoclinic, 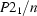

*a* = 8.4077 (9) Å
*b* = 15.5098 (16) Å
*c* = 9.5556 (10) Å
β = 94.644 (2)°
*V* = 1242.0 (2) Å^3^
*Z* = 2
Mo *K*α radiation
μ = 0.85 mm^−1^
*T* = 293 K
0.20 × 0.10 × 0.03 mm

#### Data collection

Bruker SMART CCD diffractometer
Absorption correction: none
7646 measured reflections
2442 independent reflections
1826 reflections with *I* > 2σ(*I*)
*R*_int_ = 0.057

#### Refinement

*R*[*F* ^2^ > 2σ(*F* ^2^)] = 0.046
*wR*(*F* ^2^) = 0.135
*S* = 1.06
2442 reflections
179 parameters
H-atom parameters constrained
Δρ_max_ = 0.49 e Å^−3^
Δρ_min_ = −0.59 e Å^−3^

### 

Data collection: *SMART* (Bruker, 1997 ▶); cell refinement: *SAINT* (Bruker, 1997 ▶); data reduction: *SAINT*; program(s) used to solve structure: *SHELXS97* (Sheldrick, 2008 ▶); program(s) used to refine structure: *SHELXL97* (Sheldrick, 2008 ▶); molecular graphics: *SHELXTL* (Sheldrick, 2008 ▶); software used to prepare material for publication: *SHELXTL*.

## Supplementary Material

Crystal structure: contains datablocks I, global. DOI: 10.1107/S1600536809018728/dn2454sup1.cif

Structure factors: contains datablocks I. DOI: 10.1107/S1600536809018728/dn2454Isup2.hkl

Additional supplementary materials:  crystallographic information; 3D view; checkCIF report

## Figures and Tables

**Table 1:** Hydrogen-bond geometry (Å, °)

*D*—H⋯*A*	*D*—H	H⋯*A*	*D*⋯*A*	*D*—H⋯*A*
O2—H2⋯O4	0.82	2.22	2.810 (4)	129
O1—H1*B*⋯O3	0.86	2.13	2.933 (4)	155
O1—H1*A*⋯O5^i^	0.87	2.04	2.884 (4)	165

Symmetry code: (i) 
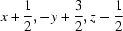
.

**Fig. 1.**
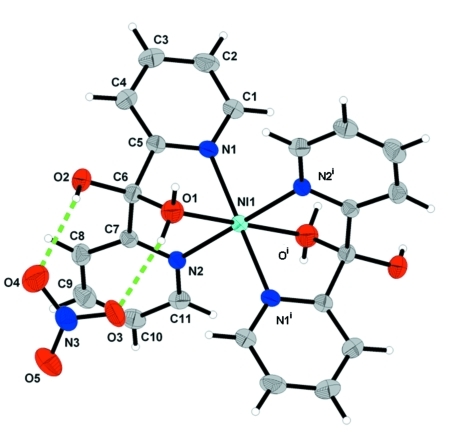
View of the title complex with the atom labeling scheme. Displacement ellipsoids are drawn at the 30% probability level. H atoms are represented as small spheres of arbitrary radii and hydrogen bonds are shown as dashed lines. [Symmetry code: (i) 1 - x, 1 - y, 1 - z].

**Fig. 2.**
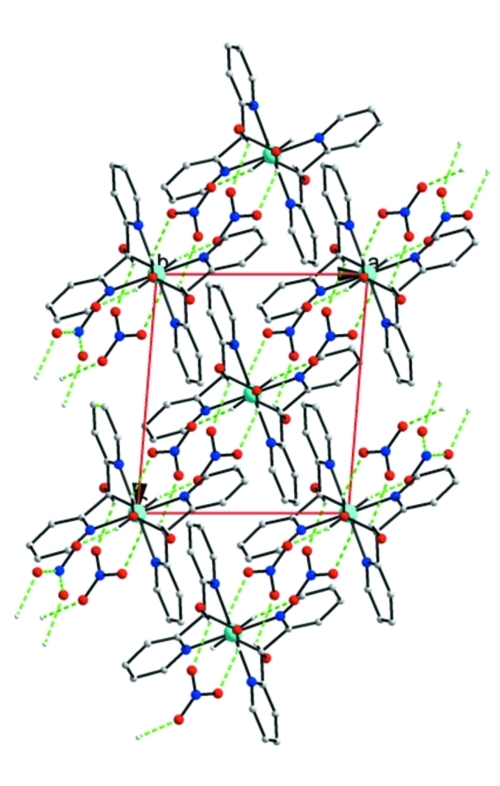
Packing view down the b axis. Hydrogen bonds are shown as dashed lines. H atoms not involved in hydrogen bondings have been omitted for clarity.

## supplementary crystallographic information

### Comment

Di-2-pyridyl ketone ((py)_2_CO) has been employed to form structurally interesting new complexes with 3 d-metal ions (Stoumpos *et al.*, 2009). Water and alcohols (ROH) have been shown to add to the carbonyl group forming the ligands (2-py)_2_C(OH)_2_ [the *gem*-diol form of (2-py)_2_CO] and (2-py)_2_C(OR)(OH) [the hemiacetal form of (2- py)_2_CO], respectively (Efthymiou *et al.*, 2006). The neutral ligands (py)_2_C(OH)_2_ and (py)_2_C(OR)(OH) coordinate to the metal centres as *N*,*N*',*O* chelates (Papaefstathiou & Perlepes, 2002). The different interesting coordination modes have been seen when the ligands (py)_2_C(OH)_2_ and (py)_2_C(OR)(OH) are deprotonated to form mono- or dianion. The deprotonation of hydroxyl groups leads to a coordinative flexibility (Papatriantafyllopoulou *et al.*, 2007; Stoumpos *et al.*, 2008). Some Ni^II^ complexes of the neutral ligand, (py)_2_C(OH)_2_ have been structuraly characterized (Wang *et al.*,  1986; Li *et al.*, 2005), but no structure with a nitrate ion as the counter-ion has been reported to date.

The Ni^II^ atom is located on an inversion center and is coordinated by two tridentate chelating (2-py)_2_C(OH)_2_ ligand to form a distorted octahedral geometry. The *gem*-diol ligand (2-py)_2_C(OH)_2_ adopts the coordination mode η^1^:η^1^:η^1^ (Fig. 1). The Ni—N and Ni—O bond lengths are typical for high-spin Ni(II) in octahedral environments [Ni—N = 2.094 (2) and 2.124 (3) Å, Ni—O = 2.108 (3) Å] (Moragues-Cánovas *et al.*, 2004). The hydrogen attached to O1 is splitting on two positions which are both in interaction with the NO_3_^-^ anion. The O—H···O hydrogen bonds build up a layer parallel to the (101) plane (Table 1, Fig. 2).

### Experimental

36.7 mg (0.125 mmol) of Ni(NO_3_)_2_.6H_2_O and 35.5 mg (0.25 mmol) of C_6_H_5_COONH_4_ were dissolved in 4 ml water and carefully layered by 4 ml solution of amixture of acetone, methanol and ethanol (2/2/2) of di-2-pyridyl ketone ligand (46.1 mg, 0.25 mmol). Suitable crystals of the title compound for X-ray analysis were obtained in a few weeks.

### Refinement

All H atoms attached to C atoms were fixed geometrically and treated as riding with C—H = 0.93 Å with U_iso_(H) = 1.2U_eq_(C). Hydroxyl H atom for O2 were treated as riding on the parent atom with O—H = 0.82 Å and U_iso_(H) = 1.5U_eq_(O). The hydroxyl H attached to O1 appears to be splitted on two positions. The coordinates of these two positions were initially refined with O—H restraints (0.85 Å) and U_iso_(H) = 1.5U_eq_(O). Then in the last stage of refinement these disordered H atoms were treated as riding on the O atom.

### Crystal data

**Table:**

[Ni(C_11_H_10_N_2_O_2_)_2_](NO_3_)_2_	*F*(000) = 604
*M**_r_* = 587.15	*D*_x_ = 1.570 Mg m^−^^3^
Monoclinic, *P*2_1_/*n*	Mo *K*α radiation, λ = 0.71073 Å
Hall symbol: -P 2yn	Cell parameters from 2352 reflections
*a* = 8.4077 (9) Å	θ = 2.5–25.6°
*b* = 15.5098 (16) Å	µ = 0.85 mm^−^^1^
*c* = 9.5556 (10) Å	*T* = 293 K
β = 94.644 (2)°	Plate, pale brown
*V* = 1242.0 (2) Å^3^	0.20 × 0.10 × 0.03 mm
*Z* = 2	

### Data collection

**Table:**

Bruker SMART CCD diffractometer	1826 reflections with *I* > 2σ(*I*)
Radiation source: fine-focus sealed tube	*R*_int_ = 0.057
graphite	θ_max_ = 26.0°, θ_min_ = 2.5°
φ and ω scans	*h* = −11→11
7646 measured reflections	*k* = −20→12
2442 independent reflections	*l* = −12→12

### Refinement

**Table:**

Refinement on *F*^2^	Primary atom site location: structure-invariant direct methods
Least-squares matrix: full	Secondary atom site location: difference Fourier map
*R*[*F*^2^ > 2σ(*F*^2^)] = 0.046	Hydrogen site location: inferred from neighbouring sites
*wR*(*F*^2^) = 0.135	H-atom parameters constrained
*S* = 1.06	*w* = 1/[σ^2^(*F*_o_^2^) + (0.0755*P*)^2^ + 0.0426*P*] where *P* = (*F*_o_^2^ + 2*F*_c_^2^)/3
2442 reflections	(Δ/σ)_max_ < 0.001
179 parameters	Δρ_max_ = 0.49 e Å^−^^3^
0 restraints	Δρ_min_ = −0.59 e Å^−^^3^

### Special details

**Table:**

Geometry. All esds (except the esd in the dihedral angle between two l.s. planes) are estimated using the full covariance matrix. The cell esds are taken into account individually in the estimation of esds in distances, angles and torsion angles; correlations between esds in cell parameters are only used when they are defined by crystal symmetry. An approximate (isotropic) treatment of cell esds is used for estimating esds involving l.s. planes.
Refinement. Refinement of *F*^2^ against ALL reflections. The weighted *R*-factor *wR* and goodness of fit *S* are based on *F*^2^, conventional *R*-factors *R* are based on *F*, with *F* set to zero for negative *F*^2^. The threshold expression of *F*^2^ > σ(*F*^2^) is used only for calculating *R*-factors(gt) *etc*. and is not relevant to the choice of reflections for refinement. *R*-factors based on *F*^2^ are statistically about twice as large as those based on *F*, and *R*- factors based on ALL data will be even larger.

### Fractional atomic coordinates and isotropic or equivalent isotropic displacement parameters (Å^2^)

**Table:**

	*x*	*y*	*z*	*U*_iso_*/*U*_eq_	Occ. (<1)
Ni1	0.5000	0.5000	0.5000	0.0334 (2)	
N1	0.3937 (3)	0.52480 (18)	0.2948 (3)	0.0389 (6)	
N2	0.2830 (3)	0.54084 (17)	0.5703 (3)	0.0347 (6)	
O1	0.5183 (3)	0.63487 (17)	0.4873 (2)	0.0574 (7)	
H1A	0.5905	0.6553	0.4370	0.086*	0.50
H1B	0.5305	0.6590	0.5688	0.086*	0.50
O2	0.3337 (3)	0.74355 (14)	0.3938 (3)	0.0539 (6)	
H2	0.3866	0.7720	0.4530	0.081*	
C1	0.3774 (4)	0.4731 (2)	0.1816 (4)	0.0478 (9)	
H1	0.4155	0.4169	0.1884	0.057*	
C2	0.3053 (5)	0.5019 (3)	0.0558 (4)	0.0639 (11)	
H2A	0.2927	0.4653	−0.0214	0.077*	
C3	0.2515 (5)	0.5865 (3)	0.0458 (4)	0.0685 (12)	
H3	0.2048	0.6076	−0.0389	0.082*	
C4	0.2677 (4)	0.6386 (3)	0.1615 (4)	0.0562 (10)	
H4	0.2310	0.6951	0.1573	0.067*	
C5	0.3394 (4)	0.6055 (2)	0.2841 (3)	0.0394 (7)	
C6	0.3583 (4)	0.6549 (2)	0.4213 (3)	0.0386 (7)	
C7	0.2392 (3)	0.61766 (19)	0.5177 (3)	0.0353 (7)	
C8	0.0989 (4)	0.6581 (2)	0.5451 (4)	0.0459 (8)	
H8	0.0700	0.7112	0.5054	0.055*	
C9	0.0030 (4)	0.6160 (3)	0.6345 (4)	0.0594 (10)	
H9	−0.0919	0.6414	0.6569	0.071*	
C10	0.0462 (4)	0.5378 (3)	0.6898 (4)	0.0521 (9)	
H10	−0.0182	0.5097	0.7500	0.063*	
C11	0.1873 (4)	0.5008 (2)	0.6552 (3)	0.0434 (8)	
H11	0.2164	0.4470	0.6916	0.052*	
N3	0.3476 (4)	0.7718 (2)	0.7595 (4)	0.0585 (8)	
O3	0.4512 (4)	0.71600 (19)	0.7535 (3)	0.0755 (9)	
O4	0.3188 (5)	0.8218 (2)	0.6577 (3)	0.1014 (12)	
O5	0.2759 (4)	0.7785 (2)	0.8634 (4)	0.0834 (9)	

### Atomic displacement parameters (Å^2^)

**Table:**

	*U*^11^	*U*^22^	*U*^33^	*U*^12^	*U*^13^	*U*^23^
Ni1	0.0362 (3)	0.0326 (3)	0.0319 (3)	0.0023 (2)	0.0050 (2)	−0.0004 (2)
N1	0.0396 (15)	0.0451 (16)	0.0326 (15)	0.0016 (11)	0.0079 (11)	−0.0016 (11)
N2	0.0340 (13)	0.0360 (15)	0.0343 (14)	0.0020 (11)	0.0047 (11)	−0.0012 (11)
O1	0.0609 (16)	0.0526 (16)	0.0587 (16)	−0.0010 (12)	0.0049 (13)	−0.0001 (12)
O2	0.0675 (17)	0.0363 (13)	0.0578 (16)	0.0069 (12)	0.0054 (12)	0.0050 (11)
C1	0.054 (2)	0.050 (2)	0.040 (2)	0.0031 (16)	0.0081 (16)	−0.0051 (16)
C2	0.084 (3)	0.072 (3)	0.036 (2)	−0.001 (2)	0.0032 (19)	−0.0137 (19)
C3	0.087 (3)	0.079 (3)	0.038 (2)	0.011 (2)	−0.004 (2)	0.006 (2)
C4	0.072 (3)	0.054 (2)	0.043 (2)	0.0102 (19)	0.0005 (18)	0.0078 (17)
C5	0.0418 (18)	0.0411 (19)	0.0363 (17)	0.0017 (14)	0.0095 (14)	0.0007 (14)
C6	0.0390 (18)	0.0349 (18)	0.0422 (18)	0.0031 (14)	0.0059 (14)	0.0046 (14)
C7	0.0365 (16)	0.0357 (17)	0.0336 (16)	0.0002 (13)	0.0026 (13)	−0.0053 (13)
C8	0.0391 (18)	0.048 (2)	0.050 (2)	0.0069 (15)	−0.0001 (15)	−0.0015 (15)
C9	0.040 (2)	0.075 (3)	0.065 (2)	0.0062 (19)	0.0128 (18)	−0.010 (2)
C10	0.047 (2)	0.064 (2)	0.047 (2)	−0.0077 (18)	0.0155 (16)	−0.0005 (18)
C11	0.0485 (18)	0.0451 (19)	0.0370 (17)	−0.0049 (16)	0.0053 (14)	0.0002 (15)
N3	0.067 (2)	0.047 (2)	0.061 (2)	−0.0010 (16)	0.0000 (17)	−0.0192 (17)
O3	0.077 (2)	0.077 (2)	0.0714 (19)	0.0337 (16)	0.0032 (15)	−0.0251 (15)
O4	0.173 (4)	0.056 (2)	0.071 (2)	0.016 (2)	−0.014 (2)	−0.0052 (16)
O5	0.071 (2)	0.092 (2)	0.092 (2)	0.0066 (16)	0.0358 (18)	−0.0195 (18)

### Geometric parameters (Å, °)

**Table:**

Ni1—N2	2.093 (2)	C2—H2A	0.9300
Ni1—N2^i^	2.093 (2)	C3—C4	1.367 (5)
Ni1—O1	2.102 (3)	C3—H3	0.9300
Ni1—O1^i^	2.102 (3)	C4—C5	1.372 (4)
Ni1—N1	2.123 (3)	C4—H4	0.9300
Ni1—N1^i^	2.123 (3)	C5—C6	1.515 (4)
N1—C5	1.334 (4)	C6—C7	1.528 (4)
N1—C1	1.345 (4)	C7—C8	1.380 (4)
N2—C7	1.333 (4)	C8—C9	1.385 (5)
N2—C11	1.340 (4)	C8—H8	0.9300
O1—C6	1.472 (4)	C9—C10	1.360 (5)
O1—H1A	0.8650	C9—H9	0.9300
O1—H1B	0.8625	C10—C11	1.382 (5)
O2—C6	1.412 (4)	C10—H10	0.9300
O2—H2	0.8200	C11—H11	0.9300
C1—C2	1.377 (5)	N3—O5	1.206 (4)
C1—H1	0.9300	N3—O3	1.233 (4)
C2—C3	1.388 (5)	N3—O4	1.253 (4)
			
N2—Ni1—N2^i^	180.0	C4—C3—C2	119.5 (4)
N2—Ni1—O1	77.70 (10)	C4—C3—H3	120.3
N2^i^—Ni1—O1	102.30 (10)	C2—C3—H3	120.3
N2—Ni1—O1^i^	102.30 (10)	C3—C4—C5	118.5 (4)
N2^i^—Ni1—O1^i^	77.70 (10)	C3—C4—H4	120.7
O1—Ni1—O1^i^	180.0	C5—C4—H4	120.7
N2—Ni1—N1	85.93 (9)	N1—C5—C4	122.7 (3)
N2^i^—Ni1—N1	94.07 (9)	N1—C5—C6	113.4 (3)
O1—Ni1—N1	78.10 (10)	C4—C5—C6	123.9 (3)
O1^i^—Ni1—N1	101.90 (10)	O2—C6—O1	113.6 (2)
N2—Ni1—N1^i^	94.07 (9)	O2—C6—C5	109.1 (3)
N2^i^—Ni1—N1^i^	85.93 (9)	O1—C6—C5	107.0 (2)
O1—Ni1—N1^i^	101.90 (10)	O2—C6—C7	112.8 (2)
O1^i^—Ni1—N1^i^	78.10 (10)	O1—C6—C7	106.4 (2)
N1—Ni1—N1^i^	180.000 (1)	C5—C6—C7	107.6 (2)
C5—N1—C1	119.1 (3)	N2—C7—C8	123.3 (3)
C5—N1—Ni1	110.9 (2)	N2—C7—C6	113.0 (2)
C1—N1—Ni1	130.0 (2)	C8—C7—C6	123.7 (3)
C7—N2—C11	118.7 (3)	C7—C8—C9	116.8 (3)
C7—N2—Ni1	111.76 (19)	C7—C8—H8	121.6
C11—N2—Ni1	129.5 (2)	C9—C8—H8	121.6
C6—O1—Ni1	99.56 (17)	C10—C9—C8	120.7 (3)
C6—O1—H1A	110.0	C10—C9—H9	119.7
Ni1—O1—H1A	117.0	C8—C9—H9	119.7
C6—O1—H1B	109.5	C9—C10—C11	119.0 (3)
Ni1—O1—H1B	112.6	C9—C10—H10	120.5
H1A—O1—H1B	107.8	C11—C10—H10	120.5
C6—O2—H2	109.5	N2—C11—C10	121.5 (3)
N1—C1—C2	121.2 (4)	N2—C11—H11	119.3
N1—C1—H1	119.4	C10—C11—H11	119.3
C2—C1—H1	119.4	O5—N3—O3	120.1 (4)
C1—C2—C3	119.0 (4)	O5—N3—O4	120.5 (4)
C1—C2—H2A	120.5	O3—N3—O4	119.4 (4)
C3—C2—H2A	120.5		

Symmetry codes: (i) −*x*+1, −*y*+1, −*z*+1.

### Hydrogen-bond geometry (Å, °)

**Table:**

*D*—H···*A*	*D*—H	H···*A*	*D*···*A*	*D*—H···*A*
O2—H2···O4	0.82	2.22	2.810 (4)	129
O1—H1B···O3	0.86	2.13	2.933 (4)	155
O1—H1A···O5^ii^	0.87	2.04	2.884 (4)	165

Symmetry codes: (ii) *x*+1/2, −*y*+3/2, *z*−1/2.
